# Navigating treatment for basidiobolomycosis: a qualitative review of 24 cases

**DOI:** 10.1186/s12879-024-09664-8

**Published:** 2024-08-12

**Authors:** Gholamreza Pouladfar, Samaneh Jahangiri, Amirhossein Shahpar, Mohsen Nakhaie, Aryan Mohamadi Nezhad, Zahra Jafarpour, Anahita Sanaee Dashti

**Affiliations:** 1 Alborzi clinical microbiology research center, department of pediatrics, School of medicine, Nemazi hospital, Shiraz University of medical sciences, Shiraz, Iran; 2 Student Research Committee, Shiraz University of Medical Sciences, Shiraz, Iran; 3 Clinical Research Development Unit, Afzalipour Hospital, Kerman University of Medical Sciences, Kerman, Iran; 4 Gastroenterology and Hepatology Research Center, Institute of Basic and Clinical Physiology Sciences, Kerman University of Medical Sciences, Kerman, Iran

**Keywords:** Basidiobolomycosis, Antifungal, Tropical diseases

## Abstract

**Background and Objectives:**

Zygomycosis, a severe form of fungal infection, is classified into two categories: Mucorales and Entomophthorales. Within the Entomophthorales category, Basidiobolomycosis is a rarely recognized genus that can have significant health implications. Prompt diagnosis and appropriate treatment, which includes the use of antifungal medication and surgical procedures, are vital for enhancing the prognosis of patients. The objective of this study is to investigate the response to treatment in patients hospitalized due to basidiobolomycosis.

**Methods:**

We carried out a retrospective study, in which we analyzed data from 49 patients who were diagnosed with Entomophthorale, Zygomycosis, and Basidiobolomycosis at Namazi Hospital, Shiraz, between the years 1997 and 2019. The data included parameters such as demographic information, clinical symptoms, imaging findings, treatment methods, and patient outcomes.

**Results:**

Out of 49 patients, 24 children, predominantly male (83.3%), were definitively diagnosed with basidiobolomycosis. The ages of the patients ranged from 1 to 16 years, with an average of 5.75 years. The most frequently observed clinical manifestations included abdominal pain (70.8%), fever (54.2%), hematochezia (41.7%), vomiting (20.8%), and anorexia (16.7%). Half of the patients exhibited failure to thrive (FTT), while abdominal distension was present in 25% of the cases, and a palpable abdominal mass was found in 37% of the patients. The primary treatment strategy incorporated surgical interventions complemented by a comprehensive antifungal regimen. This regimen included medications such as amphotericin B, cotrimoxazole, itraconazole, potassium iodide, and voriconazole. These were mainly administered in a combination therapy pattern or as a monotherapy of amphotericin B. Twenty-two patients were discharged, while two patients died due to complications from the disease.

**Conclusion:**

Our findings indicate that the prevailing treatment modalities generally involve surgical intervention supplemented by antifungal regimens, including Amphotericin B, Cotrimoxazole, Potassium Iodide, and Itraconazole.

**Supplementary Information:**

The online version contains supplementary material available at 10.1186/s12879-024-09664-8.

## Introduction

Out of the more than 100,000 species of fungi in the world, only 150 are known to cause human disease [1]. However, these 150 pathogenic fungi are responsible for the death of more than 1.5 million people and the infection of over 1 billion people globally. Despite such alarming reports, fungal diseases remain a neglected topic among health researchers [2]. Furthermore, with attainable treatments, preventing the mortality caused by fungal diseases is possible.

Zygomycosis is a class of fungal infections caused by Zygomycetes, which can be subdivided into Mucorales and Entomophthorales. Entomophthorales fungi are rare pathogens that cause subcutaneous and cutaneous infections in people with a healthy immune system. These infections are generally limited to the skin and rarely affect internal organs [1, 3].

Basidiobolomycosis is a rare fungal infection caused by Basidiobolus species, members of the order Entomophthorales. This infection primarily affects the skin and subcutaneous tissues but can also involve the gastrointestinal tract. The first culture-proven case of invasive basidiobolomycosis involving the maxillary sinus and palate was reported in the United States in 1978. This marked a significant milestone in understanding the clinical manifestations of the disease. [4]. Basidiobolomycosis predominantly affects young males and is typically transmitted through traumatic inoculation. However, recent years have seen an increase in cases involving the digestive system, affecting organs such as the stomach, liver, and intestines. The infection is more common in tropical and subtropical regions, with numerous cases reported from South America, Saudi Arabia, Australia, Egypt, Kuwait, and Iran [5]. In Iran alone, 17 new cases were reported between 2010 and 2015 [1].

The clinical presentation of basidiobolomycosis varies depending on the site of infection. Subcutaneous infections are characterized by firm, painless swellings, while gastrointestinal infections can mimic malignancies or inflammatory bowel diseases. Despite its potential severity, basidiobolomycosis is rare, and its risk factors, clinical characteristics, and treatment options remain poorly understood. Interestingly, the disease often affects immunocompetent individuals, which is unusual for fungal infections. This aspect makes the disease particularly intriguing and challenging to diagnose [5].

The optimal treatment approach for basidiobolomycosis involves a combination of surgical resection of the inflammatory masses and systemic antifungal therapy. Several antifungal agents have been used effectively in the management of this condition. Amphotericin B binds to ergosterol in the fungal cell membranes, causing ion channel formation and cell death. The recommended dosage ranges from 0.7 to 1 mg/kg/day and is reserved for select invasive fungal infections. Azoles, such as itraconazole and posaconazole, disrupt ergosterol synthesis, disturbing cell membrane integrity. Posaconazole has the broadest activity spectrum among azoles and is effective for difficult-to-treat infections. Co-trimoxazole, an affordable antibiotic, affects folate synthesis, which is necessary for DNA and protein production in microbes. It exhibits inhibitory actions against Basidiobolus. Potassium iodide (SSKI) is an effective and low-cost treatment that is easily absorbed and distributed in the body. It can be given at lower doses to reduce side effects. Among the antifungal agents, itraconazole is the most commonly used (73% of cases), followed by amphotericin B (22%). However, amphotericin B resistance has been reported, and combining amphotericin B with an azole like itraconazole is a common initial approach [6–11].

Effective treatment is crucial for patients suffering from basidiobolomycosis. Therefore, this retrospective study aims to provide valuable insights for healthcare providers and researchers by evaluating the treatment approaches used between 1997 and 2019. Specifically, the study will identify associated complications, assess response rates to various treatments, and document instances of disease recurrence. The findings will improve patient care and support future research in managing basidiobolomycosis.

## Material and method

### Study design

This retrospective cross-sectional study examined patients diagnosed with basidiobolomycosis at Namazi Hospital between 1997 and 2019. Namazi Hospital, located in the Namazi square of Shiraz in Fars Province, is the premier referral hospital in the southern region of Iran. The study collected data on demographic information, clinical and laboratory findings, and responses to various treatments. The study was granted approval by the Institutional Review Board (IR.SUMS.MED.REC.1399.057). Before the publication of this case series, informed consent was obtained from all the participants and from their legal guardians.

### Study population

In this retrospective study, we included 24 patients diagnosed with basidiobolomycosis based on defined inclusion criteria. The study population comprised patients aged ≤ 18 years who had positive pathology findings indicative of basidiobolomycosis fungal infection. These patients were identified through the hospital’s health information systems (HIS) and enrolled in the study. In our center, the diagnosis of basidiobolomycosis is based on tissue culture, which is the gold standard for diagnosis, and histopathologic features and fungal staining of the biopsy or resected lesions obtained during surgery. The key histopathologic features include mixed suppurative and granulomatous inflammation, prominent tissue eosinophilia, presence of thin-walled, broad hyphae, some of which are surrounded by an eosinophilic amorphous material (Splendore–Hoeppli phenomenon), and the presence of zygospores that closely resemble trophozoites of free-living amoebae.

To collect comprehensive patient data, we developed a detailed questionnaire that encompassed various aspects: demographic features, clinical manifestations (including fever, fever cessation time, appetite, failure to thrive (FTT) based on Gomez criteria, pallor, abdominal distension, skin rash, diarrhea, constipation, palpable abdominal mass, vomiting, jaundice, abdominal pain, bloody stools, and skin nodules), laboratory data, radiographic findings, surgical descriptions, and treatment details (including start and end times, dosage, and administration method). We also recorded the prescribed medications upon discharge and documented the patient’s outcomes, including discharge recommended by physicians, discharge upon personal consent, or death. Whenever possible, we conducted telephone follow-ups to gather additional information on the duration of outpatient follow-up, re-hospitalization, medication timelines, and the patients’ final clinical status.

### Statistical analysis

We evaluated frequency distributions for all categorical variables. Summary statistics encompassed standard deviations (SDs) and ranges for all variables. Due to the limited number of reported cases and their heterogeneity, we deemed tests of statistical significance inappropriate. The data analysis was conducted using IBM SPSS Statistics version 26 for Windows (SPSS, Inc., Chicago, Illinois, USA).

### Ethical considerations

Ethical guidelines for human subjects research were strictly followed, including obtaining ethical approval and taking measures to protect participant privacy and confidentiality.

## Result

**Fig. 1:**
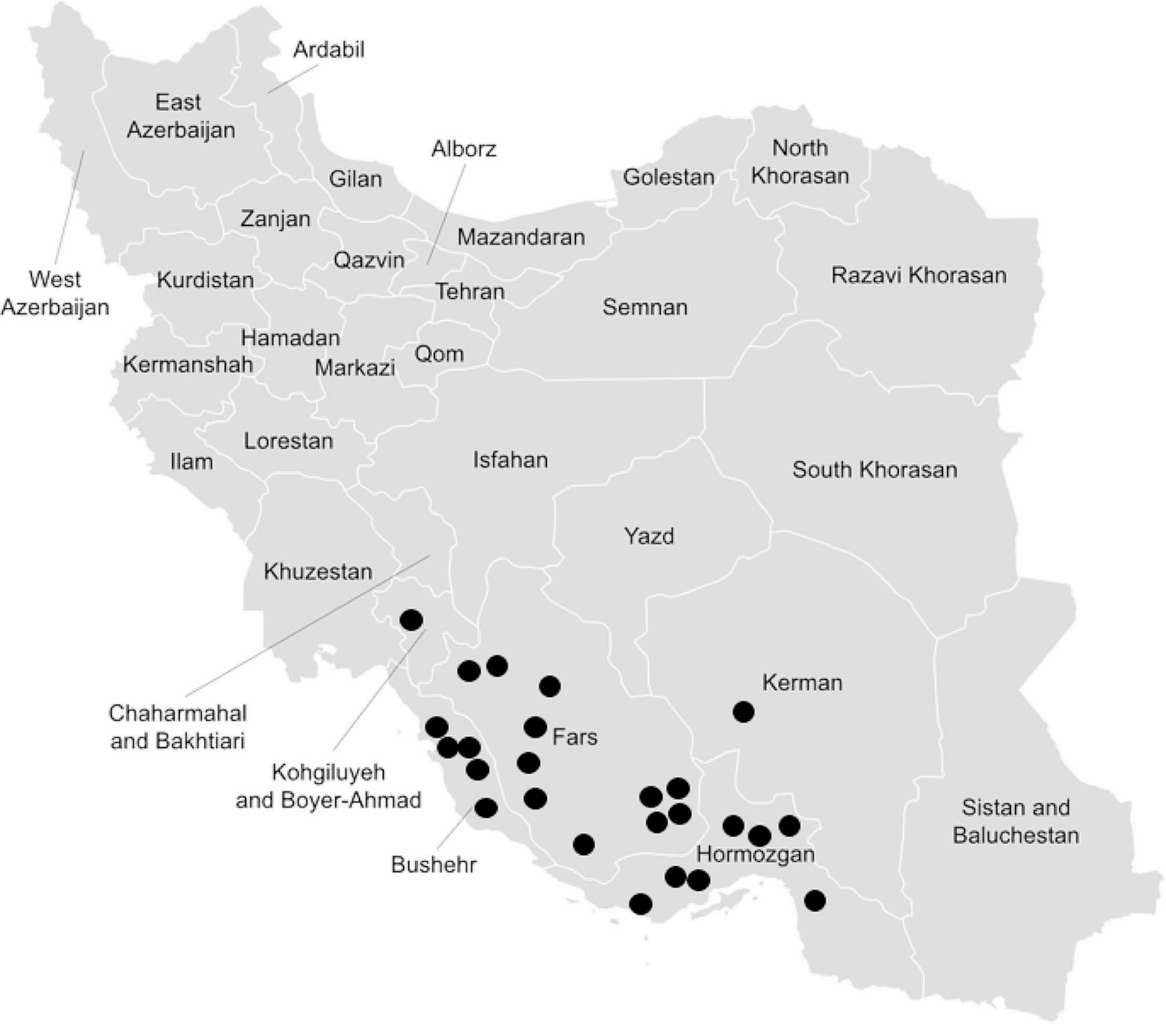
Geographic Distribution of Hospitalized Children with Basidiobolomycosis, Categorized by Province of Residence (each point represents one patient)

Among the study population, 20 children (83.3%) were male, while 4 (16.7%) were female. The mean age of the patients was 5.75 years (± 4.28 years), with an age range spanning from one year to 16 years (Fig. 1).

### Clinical manifestations

Fever was present in more than half of the patients (13, 54.2%). The duration of fever varied significantly, ranging from a minimum of one day to a maximum of 32 days following the initiation of antifungal treatment. Notably, the exact time of fever resolution was not recorded in two cases. On average, the fever subsided after 14 days (± 12 days).

In the study population, abdominal pain was the most common symptom, prompting 17 patients (70.8%) to seek medical attention. This was followed by the presence of palpable abdominal masses in 9 patients (37.5%) and blood in stools reported by 10 patients (41.7%). Abdominal distension affected 6 patients (25%). Nausea and vomiting were experienced by 5 patients (20.8%), and an equal percentage of patients also had diarrhea. Appetite loss was reported in 4 patients (16.7%), while constipation was the least common symptom, occurring in only 3 cases (Table 1).

**Table 1:** Clinical symptoms and lesion characteristics

Characteristic	Value
**Clinical Signs and Symptoms**	
Abdominal Pain	17/24 (70.8%)
Fever	13/24 (54.2%)
FTT^*^	12/22 (54.5%)
Hematochezia	10/24 (41.7%)
Palpable Abdominal Mass	9/24 (37.5%)
Abdominal Distention	6/24 (25%)
Diarrhea	5/24 (20.8%)
Vomiting	5/24 (20.8%)
Loss of Appetite	4/24 (16.7%)
Constipation	3/24 (12.5%)
Pallor	2/24 (8.3%)
Jaundice	1/24 (4.2%)
Rash	0/24 (0%)
Skin Nodule	0/24 (0%)
**Lesions Characteristic**	
Lymph Node in Ultrasound(mm)	16 (7)
Mass in Ultrasound(mm)	55 (31.6)
Mass in CT Scan(mm)	69 (40)

Data are the proportion (%) of patients for whom data were available or mean (±SD)

*A total of 22 patients were considered for the analysis of FTT due to the unavailability of data for 2 cases

FTT: Failure to Thrive

Regarding skin findings, no cases of rash or skin nodules were observed; only 3 cases exhibited jaundice. Except for two instances where failure to thrive (FTT) could not be assessed due to incomplete records, half of the studied individuals met the criteria for FTT based on the Gomez weight-for-age table. A comprehensive report of the laboratory data is presented in Table 2.

**Table 2:** Characterization of laboratory parameters in basidiobolomycosis patients

Laboratory test	Value Range	Mean (SD)	Median
CRP at Admission (mg/L)	1-150	55.37(53.00)	30
CRP at Discharge/Hospitalization (mg/L)	1-120	23.87(36.00)	9
CRP Normalization Time (days)	15–54	29.83(13.00)	30
ESR at Admission (mm/h)	1-126	50.00 (32.85)	56.5
ESR at Discharge/Hospitalization (mm/h)	7–94	34.00 (26.46)	25
ESR Normalization Time (days)	20–40	30.00 (10.00)	30
WBC at Admission (cells/µL)	6500–31,500	18,387 (6,950)	19,200
WBC at Discharge/Hospitalization (cells/µL)	5400–20,400	10,320 (3,900)	9600
Hemoglobin at Admission (g/dL)	7.3–13.1	10.40 (1.60)	10.5
Neutrophils at Admission (%)	35-84%	60.76% (15.66%)	60%
Neutrophils at Discharge/Hospitalization (%)	26-95%	55.21% (19.37%)	53%
Eosinophils at Admission (%)	1-17%	9.70% (4.90%)	9.5%
Eosinophils at Discharge/Hospitalization (%)	2-17%	9.70% (4.98%)	9.5%
Lymphocytes at Admission (%)	7.5-56.9%	26.21% (13.61%)	22%
Lymphocytes at Discharge/Hospitalization (%)	1.7-67%	32.62% (17.78%)	34%
Monocytes at Admission (%)	1-10%	5.40% (3.60%)	5%
Monocytes at Discharge/Hospitalization (%)	4-9%	6.40% (1.81%)	6%
Mixed WBC (%)	4.7-15.2%	8.27% (3.84%)	7.1%
Lowest Hemoglobin during Hospitalization (g/dL)	5.6–11.5	8.70 (1.30)	8.7
Highest Platelet Count (cells/µL)	274–994	640,170 (193,360)	638,500
Creatinine Increase during Treatment (%)	0-133%	23.39% (34.26%)	0%
BUN at Admission (mg/dL)	4–27	9.00 (5.32)	7.5
BUN at Discharge/Hospitalization (mg/dL)	1–82	14.52 (15.64)	13
SGOT (U/L)	12–125	30.00 (28.00)	19
SGPT (U/L)	5-152	23.00 (37.00)	12
Alkaline Phosphatase (U/L)	174–1470	399.00 (332.00)	276
Sodium (mmol/L)	130–144	136.60 (3.80)	137

CRP: C-reactive protein, ESR: Erythrocyte sedimentation rate, WBC: White blood cells, BUN: Blood urea nitrogen, SGOT: Serum glutamic-oxaloacetic transaminase, SGPT: Serum glutamic-pyruvic transaminase

### Gastrointestinal involvement

Based on clinical manifestations, large intestine involvement was predominant, observed in 17 cases (70.8%). Other manifestations included liver involvement in 2 cases (8.3%), small intestine involvement in 2 cases (8.3%), ophthalmic involvement in 2 cases (8.3%), and gastric involvement in 1 case (4.2%).

### Lesions and masses

All patients exhibited lesions or masses during radiographic or surgical evaluations. The number of lesions or masses varied among the patients, ranging from one to five. A single lesion was present in 21 patients (87.5%). Multiple masses were observed in a few cases: two masses in one case, three masses in one case, and five masses in one case. The size of the lesions ranged from 1 cm to 12 cm, with the most common size being approximately 4 cm.

### Imaging findings

Among the 6 cases with computed tomography (CT) scan reports, the dimensions of the lesions ranged from 27 mm to 150 mm, with a mean size of 69 mm (± 40 mm). Conversely, the lesion sizes among the 15 patients with ultrasound reports varied between 10 mm and 120 mm, with a mean length of 55 mm (± 31.6 mm) (Table 1).

### Lymph node involvement

Enlarged lymph nodes around the site of infection were observed in 10 patients (41.6%). The size of these lymph nodes ranged from 6 mm to 27 mm, with a mean size of 16 mm (± 7 mm). These findings were reported through ultrasound or CT scans or discovered during surgery (Table 1).

### Antifungal treatment regimens

Over the 22-year study period, various antifungal regimens were employed among the patients. All treatment plans included amphotericin B, ensuring every patient received this medication. One case experienced an unknown interruption in treatment, while the remaining patients received the drug until discharge. The most frequently used therapy consisted of a three-drug combination: amphotericin B, cotrimoxazole, and potassium iodide, administered to approximately 40% of the patients (10 individuals). Another three-drug combination, consisting of amphotericin B, cotrimoxazole, and itraconazole, was used in 5 cases (20%). Additionally, 2 patients received amphotericin B monotherapy, while others received either two-drug or five-drug regimens (Table 3). The mean duration of antifungal treatment with amphotericin B was 33.17 days (± 14.42 days). For cotrimoxazole, the mean duration was 28.47 days (± 16.12 days). Itraconazole had a mean treatment duration of 19.1 days (± 16 days). The mean duration of potassium iodide treatment was 31.67 days (± 11.67 days) (Table 4). Detailed information regarding the dosage and route of administration for each antifungal regimen is provided in Table 5.

**Table 3:** Antifungal regimens in patients

Antifungal Regimen	Patients (%)
Amphotericin + Cotrimoxazole + Potassium Iodide	10 (41.7%)
Amphotericin + Cotrimoxazole + Itraconazole	5 (20.8%)
Amphotericin + Itraconazole	1 (4.2%)
Amphotericin	2 (8.3%)
Amphotericin + Itraconazole + Potassium Iodide	1 (4.2%)
Amphotericin + Cotrimoxazole	1 (4.2%)
Amphotericin + Cotrimoxazole + Itraconazole + Potassium Iodide	2 (8.3%)
Amphotericin + Cotrimoxazole + Itraconazole + Potassium Iodide + Voriconazole	1 (4.2%)
Amphotericin + Potassium Iodide	1 (4.2%)

**Table 4:** Medication usage statistics and duration in patients

Medication	Frequency (%)	Duration range (days)	Mean Duration (SD)	Median Duration (days)	Mode Duration (days)
**Antifungals**					
Amphotericin	24 (100%)	7–77	33.17 (14.42)	30	30
Cotrimoxazole	19 (79.2%)	6–77	28.47 (16.12)	30	30
Itraconazole	10 (41.7%)	3–47	19.10 (16.00)	30	15
Potassium Iodide	15 (62.5%)	15–50	31.67 (11.67)	30	-
Voriconazole	1 (4.2%)	3–3	3.00 (0)	3	3
**Antibiotics**					
Ceftriaxone	11 (45.8%)	4–44	19.36 (13.34)	15	15
Metronidazole	15 (62.5%)	2–57	17.40 (15.40)	15	15
Vancomycin	6 (25%)	11–33	19.83 (9.90)	14.5	14
Meropenem	7 (29.2%)	15–38	27.86 (9.19)	32	15, 32
Imipenem	1 (4.2%)	11–11	11.00 (0)	11	11
Erythromycin	3 (21%)	4–10	6.33 (3.21)	5	4, 5, 10
Ciprofloxacin	2 (8.3%)	2–15	8.50 (9.19)	8.5	2, 15

**Table 5:** Pharmacotherapeutic administration details for selected antimicrobial agents

Antimicrobial Agent	Route of Administration	Dosage	Dosing Interval	Special Considerations
Trimethoprim/Sulfamethoxazole (TMP/SMX)¹	Intravenous	8–12 mg/kg/day (TMP component)	q12h	Maximum: 160 mg TMP/dose
Liposomal Amphotericin B	Intravenous infusion	3–5 mg/kg/dose	q24h	-
Potassium Iodide (KI)²	Oral	30 mg/kg/day	q24h	-
Itraconazole	Oral	10 mg/kg/day	q12h	-
Voriconazole³	Intravenous	LD: 9 mg/kg/dose, MD: 8 mg/kg/dose	q12h	LD for initial 24 h; MD thereafter

LD = Loading Dose; MD = Maintenance Dose; q12h = every 12 h; q24h = every 24 h

^1^TMP/SMX dosing is based on the trimethoprim component

^2^Potassium Iodide is administered as a saturated solution (SSKI), with a concentration of 325 mg per 5 ml oral solution

^3^Voriconazole requires a loading dose for the first 24 h, followed by a maintenance dose

### Antibiotic therapy

In addition to antifungal medications, antibiotic therapy was also a critical component of the treatment regimen for the patients. Ceftriaxone was administered to 11 patients (45.8%), with a mean duration of 19.36 days (± 13.34 days). Metronidazole was prescribed to 15 patients (62.5%), with a mean treatment duration of 17.4 days (± 15.4 days). Other commonly used antibiotics included vancomycin, which was prescribed to 6 patients (25%), and meropenem, administered to 7 patients (29.2%). The mean duration of vancomycin therapy was 19.83 days (± 9.9 days), while the mean duration for meropenem was 9.19 days (± 27.86 days). Imipenem was used in one case, although no further information regarding its duration or other details was provided (Table 4).

### Outcome

In our study of 24 hospitalized patients, 22 (91.7%) were successfully discharged. Regrettably, one patient (4.2%) died during the treatment period. Another patient (4.2%) initially responded well to the treatment but ultimately died after two months of oral antifungal therapy.

Among the patients with available follow-up data, nine individuals continued antifungal therapy for a mean duration of 3.6 months (± 2.1 months) until achieving complete recovery without any disease recurrence. Notably, they could resume normal daily activities without experiencing any adverse effects from either the disease or the treatment. Additionally, one patient who had received inpatient treatment for approximately two months was in complete remission at the time of data collection.

## Discussion

The class Zygomycetes consists of two orders, namely, Mucorales and Entomophthorales. Basidiobolus ranarum belongs to the Entomophthorales order and primarily causes slow progressive cutaneous and subcutaneous infections. Lesions present as firm, painless, erythematous plaques with or without ulceration, predominantly affecting individuals with a healthy immune system [12, 13]. Gastrointestinal basidiobolomycosis is a rare condition increasingly reported recently [14]. A comprehensive study conducted in 2012 showed that only 44 cases of gastrointestinal basidiobolomycosis had been reported worldwide until 2010 [15]. However, recent studies indicate an increasing trend in basidiobolomycosis cases in many regions [16]. This highlights the need for more research on the clinical features and the most appropriate management approach to reduce mortality and morbidity.

This retrospective study aims to investigate the clinical characteristics, treatment methods, and outcomes of patients with basidiobolomycosis over 22 years at Namazi Hospital in Shiraz. Our study shows that this infection is more prevalent among young males, particularly those residing in tropical regions, which aligns with the known demographic distribution of this tropical disease from previous studies [16, 17].

We observed that the most common clinical symptoms of basidiobolomycosis were abdominal pain (70.8%) and fever (54.2%). These symptoms are in line with the invasive nature of the condition. They are nearly consistent with previous reports, which also identified abdominal pain as the most common symptom, with an occurrence rate between 80% and 100% [16, 18–20]. Notably, 37% of the patients in our study had a palpable abdominal mass, which is consistent with the previous report, which found that nearly 30% of patients had a similar physical examination [19]. This underscores the importance of physical examination in the diagnostic process. Furthermore, in conjunction with the physical examination, the most common findings on CT scan and ultrasound were the presence of an abdominal mass, which was observed in nearly all cases, according to radiographic reports.

Gastrointestinal basidiobolomycosis is a rare condition [14]. Our study revealed that only two cases had ocular involvement, while the rest had gastrointestinal basidiobolomycosis. The large intestine was the most commonly affected site, accounting for 70.8% of cases, followed by the liver and small intestine with 8.3% each. This is consistent with a previous review, which also identified the colon and rectum as the most common site for gastrointestinal basidiobolomycosis, accounting for 82% of cases [15].

The treatment regimens to manage this complex infection include Amphotericin B, cotrimoxazole, itraconazole, potassium iodide, and voriconazole, with or without antibiotics to control superinfections. Amphotericin B was the most commonly used medication in almost all cases (100%), followed by itraconazole and potassium iodide, with a usage rate of 79.2% and 62.5%, respectively. Our study shows that a combination of antifungal therapy, mainly Amphotericin B plus cotrimoxazole plus potassium iodide, along with surgical intervention, can lead to favorable outcomes, indicated by the high discharge rate of 91.7%. Indeed, the use of combination therapy in our patients aligns with previous studies that underscore the efficacy of combination therapy as the optimal treatment choice [19–23]. Contrary to our study, which reported a 100% usage rate of Amphotericin B for the treatment of basidiobolomycosis, many previous studies have demonstrated a high failure rate following the use of Amphotericin B. Most of these studies advocate using Itraconazole as the superior antifungal treatment [4, 9, 15, 24, 25]. It’s noteworthy that, except for two cases, all of our cases received combined therapy. Therefore, it’s impossible to identify the best drug of choice. Moreover, in our study, all patients except for the two cases with ocular involvement underwent surgical procedures. These surgeries were primarily performed for diagnostic purposes, such as obtaining tissue samples for histopathological examination and culture. However, they also contributed significantly to the overall treatment strategy by debulking the lesions, removing necrotic tissue, and facilitating the penetration of antifungal agents into the affected areas. It is important to emphasize that surgery is a critical component of the comprehensive treatment approach for basidiobolomycosis However, the mortality rate of 8.3% indicates the need for continued research into more effective treatment protocols and early intervention strategies. Future studies should focus on the long-term follow-up of discharged patients to assess the recurrence rate and the efficacy of outpatient drug therapy. Complete recovery, characterized by the disappearance of patients’ symptoms, has been reported to commence between 8 and 9 months in previous studies [19, 22]. In our study, the inpatient treatment duration ranged from three weeks to two months, and the outpatient treatment predominantly lasted between three and six months. This indicates a total treatment duration of five to nine months. Notably, in our study and all the review studies we referenced, no recurrence of the condition has been reported.

Given the rarity of basidiobolomycosis and the challenges associated with its diagnosis, especially in resource-limited settings, we propose a set of major and minor criteria based on our findings and the existing literature. These criteria aim to facilitate early recognition and timely intervention, particularly in areas where advanced diagnostic techniques may not be readily available.Major Criteria:

1. Histopathological findings: Presence of broad, sparsely septate hyphae surrounded by eosinophilic material (Splendore-Hoeppli phenomenon).
2. Positive culture for Basidiobolus species.
3. Identification of Basidiobolus DNA through molecular techniques (e.g., PCR).

Minor Criteria:

1. Clinical presentation: (a) Abdominal pain (present in 70.8% of our cases) (b) Fever (54.2% of cases) (c) Hematochezia (41.7% of cases) (d) Palpable abdominal mass (37% of cases).
2. Demographic factors: (a) Young age (mean age 5.75 years in our study) (b) Male predominance (83.3% in our cohort).
3. Imaging findings: (a) Presence of abdominal mass on CT or ultrasound (b) Thickening of the bowel wall, particularly in the colon.
4. Laboratory findings: (a) Peripheral eosinophilia (b) Elevated inflammatory markers (e.g., CRP, ESR).
5. Geographical consideration: a. Residence in or recent travel to endemic areas (tropical and subtropical regions).

We suggest that a definitive diagnosis of basidiobolomycosis could be made with either one major criterion or three minor criteria plus a positive response to antifungal therapy. This approach allows for a more accessible diagnostic pathway in resource-limited settings where advanced molecular or histopathological techniques may not be available.

However, it is crucial to emphasize that whenever possible, obtaining tissue for histopathological examination and culture remains the gold standard for a definitive diagnosis. The proposed criteria should be used as a guide to raise clinical suspicion and initiate appropriate investigations or empirical treatment when necessary.

### Limitations

This study has some limitations that need to be acknowledged. First, the retrospective design may have resulted in limitations in data collection. Second, the study was conducted at a single center, which may restrict the applicability of the findings to a broader context. Third, the use of combined therapy makes it difficult to determine the most effective drug for treating basidiobolomycosis. Lastly, the lack of complete follow-up for all patients is a limitation. Future research should focus on conducting prospective, multicenter studies to validate our findings and determine the most effective treatment for this condition. Despite these limitations, this study’s findings contribute to the growing knowledge of basidiobolomycosis and its treatment.

## Conclusion

This investigation underscores the clinical importance of basidiobolomycosis, a fungal infection primarily affecting young males in tropical locales. The presentation of this disease is typically characterized by symptoms such as abdominal pain, fever, abdominal masses, and bloody diarrhea. Our findings indicate that the prevailing treatment modalities generally involve surgical intervention supplemented by a comprehensive antifungal regimen, including Amphotericin B, Cotrimoxazole, Potassium Iodide, and Itraconazole. Treatment durations have varied, extending from one month of inpatient care to six months of outpatient management, with most cases achieving complete recovery.

### Electronic supplementary material

Below is the link to the electronic supplementary material.

Supplementary Material 1

## Data Availability

The datasets used and/or analyzed during the current study are available from the corresponding author on reasonable request.
