# Supplementary material for: Navigating treatment for basidiobolomycosis: a qualitative review of 24 cases
Source: BMC Infect Dis. 2024 Aug 12;24:816. doi: 10.1186/s12879-024-09664-8 (PMC11318116; doi:10.1186/s12879-024-09664-8)
Supplement: Supplementary file 1 — Supplementary Material 1 [file 12879_2024_9664_MOESM1_ESM.docx]

**Questionnaire**

| **Demographic Information** | | | |
| --- | --- | --- | --- |
| Name: | Age: | | Gender: M / F |
| Hospitalization code: | | File Number: | |

| **Clinical Presentation** | |
| --- | --- |
| Date of hospitalization: __ /__ /__ | Discharge date: __ /__ /__ |
| Date of death (if applicable): __ /__ /__ | Number of hospitalizations: ____ |

| **Presenting symptoms (check all that apply):** | | | |
| --- | --- | --- | --- |
| ▢ Fever | ▢ Anorexia | ▢ Failure to thrive (FTT) | ▢ Constipation |
| ▢ Fever | ▢ Anorexia | ▢ Failure to thrive | ▢ Palpable abdominal mass |
| ▢ Pallor | ▢ Abdominal distension | ▢ Diarrhea | ▢ Vomiting |
| ▢ Hematochezia | ▢ Jaundice | ▢ Abdominal pain | ▢ Other: ___________ |

| **Gastrointestinal involvement (check all that apply):** | | | | |
| --- | --- | --- | --- | --- |
| ▢ Large intestine | ▢ Small intestine | ▢ Liver | ▢ Stomach | ▢ Other: |

| **Diagnostic Imaging** | |
| --- | --- |
| Ultrasound findings: |  |
| CT scan findings: |  |
| Number of lesions/masses: ______ | Size range of lesions/masses: ______ cm to ______ cm |
| Lymph node involvement: ▢ Yes ▢ No | Size range of enlarged lymph nodes: _____ mm to _____ mm |

| **Pathological diagnosis:** |
| --- |
|  |

| **Antifungal therapy:** | | | | | |
| --- | --- | --- | --- | --- | --- |
| Drug name | Dose | Route | Start date | Stop date | Duration (days) |
|  |  |  |  |  |  |
|  |  |  |  |  |  |
|  |  |  |  |  |  |
|  |  |  |  |  |  |

| **Antifungal therapy:** | | | | | |
| --- | --- | --- | --- | --- | --- |
| Drug name | Dose | Route | Start date | Stop date | Duration (months) |
|  |  |  |  |  |  |
|  |  |  |  |  |  |
|  |  |  |  |  |  |
|  |  |  |  |  |  |

| **Surgical intervention: ▢ Yes ▢ No** |
| --- |
| Description: |

**Time to resolution of symptoms after initiating treatment: _____ days**

**Outcome: Discharged ▢ Deceased ▢ Other: ________**

**Follow-up: Duration of outpatient follow-up: _____ months**

| **Antifungal therapy post-discharge:** | | | | | |
| --- | --- | --- | --- | --- | --- |
| Drug name | Dose | Route | Start date | Stop date | Duration (days) |
|  |  |  |  |  |  |
|  |  |  |  |  |  |
|  |  |  |  |  |  |
|  |  |  |  |  |  |

**Disease recurrence: ▢ Yes ▢ No**

| **Laboratory Investigations** | | | |
| --- | --- | --- | --- |
| Parameter | Admission | During treatment | Discharge |
| WBC |  |  |  |
| Hemoglobin |  |  |  |
| Platelets |  |  |  |
| AST |  |  |  |
| ALT |  |  |  |
| Alkaline phosphatase |  |  |  |
| OB |  |  |  |
| OP |  |  |  |
| ESR |  |  |  |
| CRP |  |  |  |
